# The RESCUE7 Framework: A Practical Mindset for Junior Physicians in the Emergency Department

**DOI:** 10.7759/cureus.101221

**Published:** 2026-01-10

**Authors:** So Sakamoto

**Affiliations:** 1 Department of Emergency Medicine, Asahi General Hospital, Asahi, JPN

**Keywords:** clinical reasoning, emergency medicine resident, medical resident education, professional identity formation, resident-training, uncertainty tolerance

## Abstract

Junior physicians working in the emergency department (ED) must manage multiple undifferentiated patients under time pressure, frequent interruptions, and high cognitive load. Difficulties in this setting rarely stem from lack of factual knowledge alone; rather, they arise due to uncertainty about how to think, prioritize, communicate, and act in real time under pressure. This editorial introduces RESCUE7, a practical mindset framework designed to make implicit supervisory expectations explicit and to support early-career physicians during initial ED exposure. RESCUE7 is not intended as an assessment scale or checklist but as a compact shared vocabulary that facilitates coaching, reflection, and real-time discussion of clinical reasoning, prioritization, and situational awareness. By externalizing tacit expert thinking into seven observable and interrelated domains, RESCUE7 aims to support safer early practice and more effective educational conversations in emergency medicine.

## Editorial

Junior physicians (including resident physicians and recently graduated doctors in their early years of independent clinical practice) working in the emergency department (ED) face a uniquely challenging learning environment. They are required to manage multiple patients simultaneously, often with incomplete information, frequent interruptions, and evolving clinical priorities. In such settings, difficulty rarely stems from lack of factual knowledge alone. Rather, it reflects uncertainty about how to think, prioritize, communicate, and act under pressure, a pattern described in the professional identity formation and diagnostic reasoning literature [1,2].

While medical education has often prioritized knowledge and procedures, competency-based medical education emphasizes integrated capabilities demonstrated in real clinical practice through assessment, feedback, and progressive entrustment. Transitions into postgraduate training are associated with heightened uncertainty, stress, and challenges in prioritization and decision-making [3,4]. These difficulties reflect not only limited experience but also challenges in tolerating uncertainty and navigating unfamiliar professional roles, particularly during early ED exposure [1,5,6].

To address this gap, I propose RESCUE7, a practical mindset framework designed to make these implicit expectations explicit. RESCUE7 is intentionally not designed as an assessment scale, behavioral checklist, or performance metric. Instead, it functions as a compact shared vocabulary that enables supervisors and junior physicians to discuss clinical reasoning, prioritization, and situational awareness in real time. In this sense, RESCUE7 complements existing frameworks such as non-technical skills and crisis resource management by serving as a conversation tool for coaching and reflection during early ED exposure.

RESCUE7 was developed through longitudinal supervisory observations of junior physicians working in ED settings. While supervising residents and early-career doctors during routine clinical shifts, case discussions, and post-shift debriefings, recurring patterns of difficulty were noted, not primarily in factual knowledge, but in real-time prioritization, anticipation, escalation, and coordination under pressure. The seven domains were iteratively identified and refined by comparing these patterns across different clinical contexts and repeatedly using the framework as a shared language during supervision and reflection. This process emphasized practical usefulness and communicability rather than formal assessment or measurement.

RESCUE7 consists of seven interrelated mindset domains: Reliance (adaptive help-seeking, including when and whom to ask, how to convey concern, and what contextual information to share); Emotional control (maintaining cognitive clarity under stress and uncertainty); Sound knowledge (reliable application of core clinical fundamentals); Clinical imagination (contextual imagination extending beyond biomedical inference); Urgency management (recognizing evolving clinical threat rather than reacting to isolated findings); Expression skills (structured, concise communication within multidisciplinary teams); and 7th gear (sustainable endurance, maintaining safe performance under prolonged workload and cognitive fatigue).

The RESCUE7 domains are not intended to function as independent competencies or discrete behavioral categories. Rather, they represent mutually reinforcing and observable tendencies that often overlap in real clinical practice. For example, effective help-seeking (Reliance) is closely linked to how concerns are framed and communicated (Expression skills), while emotional control under stress can influence urgency management and clinical imagination through attentional narrowing and cognitive load. These overlaps are expected and clinically meaningful; in practice, they provide opportunities for targeted coaching and reflection rather than indicating conceptual ambiguity within the framework.

As a minimal implementation example, RESCUE7 can be used as a set of brief coaching prompts during routine post-shift debriefs. Supervisors may ask: Which RESCUE7 domain felt most challenging during this shift? Where did you recognize uncertainty, and how did that shape your decisions? At what point did you rely on others, and how did you frame that request? Such prompts typically require only a few minutes and help make tacit expectations explicit without converting the framework into a checklist or assessment tool.

In practice, RESCUE7 can be flexibly integrated into everyday educational moments. During orientation check-ins, supervisors may use the framework to set shared expectations around prioritization, help-seeking, and pacing in the ED. In end-of-shift micro-debriefs, specific domains can be referenced to discuss observable behaviors, common pitfalls, and short coaching prompts. Similarly, during case discussions, RESCUE7 provides a shared language to reflect on mindset and decision-making processes without reducing complex clinical performance to checklist items.

By emphasizing thinking processes rather than task completion, RESCUE7 supports a shift from retrospective critique toward prospective coaching. For junior physicians, this shared vocabulary can normalize uncertainty as an inherent feature of emergency practice rather than a personal deficiency. For supervisors, it offers a concise way to articulate expectations, provide feedback, and support adaptive growth under real-world conditions.

RESCUE7 is derived primarily from supervisory observations by a single educator across multiple ED settings. Although the framework reflects recurrent patterns observed across varied contexts, it has not undergone formal empirical validation. It represents an interpretive synthesis rather than a systematically developed theory. Future research using qualitative interviews, multi-site consensus methods, and longitudinal outcome studies is needed to refine and validate the framework and to explore its impact on learning and clinical performance.

Effective ED practice requires not only clinical competence but also a mindset capable of navigating uncertainty, managing urgency, and understanding patients within their lived contexts. RESCUE7 offers a structured articulation of these expectations. By naming and normalizing the attitudes that support safe early practice, the framework may help junior physicians transition more confidently into emergency care and provide educators with a practical tool for supporting clinical reasoning and professional development.
